# Early predictors of mortality in parkinsonism and Parkinson disease: A population-based study

**DOI:** 10.1212/WNL.0000000000006576

**Published:** 2018-11-27

**Authors:** David Bäckström, Gabriel Granåsen, Magdalena Eriksson Domellöf, Jan Linder, Susanna Jakobson Mo, Katrine Riklund, Henrik Zetterberg, Kaj Blennow, Lars Forsgren

**Affiliations:** From the Department of Pharmacology and Clinical Neuroscience (D.B., M.E.D., J.L., L.F.), Epidemiology and Global Health Unit, Department of Public Health and Clinical Medicine (G.G.), Department of Psychology (M.E.D.), and Department of Radiation Sciences, Diagnostic Radiology and Umeå Center for Functional Brain Imaging (S.J.M., K.R.), Umeå University; Institute of Neuroscience and Physiology (H.Z., K.B.), Department of Psychiatry and Neurochemistry, Sahlgrenska Academy at University of Gothenburg, Mölndal; Clinical Neurochemistry Laboratory (H.Z., K.B.), Sahlgrenska University Hospital, Mölndal, Sweden; Department of Molecular Neuroscience (H.Z.), University College London Institute of Neurology; and UK Dementia Research Institute at UCL (H.Z.), London, UK.

## Abstract

**Objective:**

To examine mortality and associated risk factors, including possible effects of mild cognitive impairment, imaging, and CSF abnormalities, in a community-based population with incident parkinsonism and Parkinson disease.

**Methods:**

One hundred eighty-two patients with new-onset, idiopathic parkinsonism were diagnosed from January 2004 through April 2009, in a catchment area of 142,000 inhabitants in Sweden. Patients were comprehensively investigated according to a multimodal research protocol and followed prospectively for up to 13.5 years. A total of 109 patients died. Mortality rates in the general Swedish population were used to calculate standardized mortality ratio and expected survival, and Cox proportional hazard models were used to investigate independent predictors of mortality.

**Results:**

The standardized mortality ratio for all patients was 1.84 (95% confidence interval 1.50–2.22, *p* < 0.001). Patients with atypical parkinsonism (multiple system atrophy or progressive supranuclear palsy) had the highest mortality. In early Parkinson disease, a mild cognitive impairment diagnosis, freezing of gait, hyposmia, reduced dopamine transporter activity in the caudate, and elevated leukocytes in the CSF were significantly associated with shorter survival.

**Conclusion:**

Although patients presenting with idiopathic parkinsonism have reduced survival, the survival is highly dependent on the type and characteristics of the parkinsonian disorder. Patients with Parkinson disease presenting with normal cognitive function seem to have a largely normal life expectancy. The finding of a subtle CSF leukocytosis in patients with Parkinson disease with short survival may have clinical implications.

In Parkinson disease (PD), the second most common neurodegenerative disorder, life expectancy is reduced.^1^ There are, however, conflicting data regarding the size of, and specific factors accounting for the reduced survival in comparison to the general population. Most studies of survival in PD have been hospital-based or have used register-based case-finding methods. These designs may produce biased results, through underrepresentation of mild PD cases and lack of referral of older patients to hospital clinics.^2^ There are also few studies of the survival in unselected populations of patients with new-onset idiopathic parkinsonism (including atypical parkinsonism), rather than PD.

In PD, previous studies have found that a nontremor-dominant phenotype, PD dementia (PDD), and early autonomic dysfunction are associated with a shorter survival.^1,3,4^ The recently defined diagnosis of mild cognitive impairment in PD (PD-MCI)^5^ has rarely been studied in this regard. Furthermore, the neurobiology of PD with short survival (in terms of factors such as striatal dopamine depletion patterns, CSF abnormalities, or *APOE* genotype) is not well known.

Against this background, we assessed all-cause mortality and associated risk factors in a population-based, Swedish cohort of patients with incident, carefully diagnosed idiopathic parkinsonism, including PD. The patients underwent extensive neurologic, neuropsychological, and laboratory testing as well as multimodal neuroimaging and received standard, or if indicated, advanced treatments by movement disorder neurologists during long-term follow-up.

## Methods

### Study population

All participants were part of a population-based incidence study of unselected cases of new-onset idiopathic parkinsonism, from a defined geographic catchment area of 142,000 inhabitants in northern Sweden.^6^ The local tradition is to refer all patients with suspected PD to the Department of Neurology at Umeå University Hospital (our department), which is the only neurologic department in the area. To avoid selection bias, and to make case identification as complete as possible, a population screening was performed through many sources, including letters sent twice yearly to all health practitioners asking for referral of all suspected cases with incident parkinsonism. Eldercare institutions were surveyed by visits (the largest institution) with an examination by neurologists of all cases that were reported to have signs of parkinsonism, or by interview (remaining institutions).

All patients were recruited to the study in the early motor (drug-naive) phase, between January 1, 2004, and April 30, 2009. After exclusion of patients with secondary parkinsonism (e.g., due to neuroleptic drugs or stroke) or dementia at baseline (e.g., patients with dementia with Lewy bodies), 182 participants were included and followed prospectively. A diagnosis of PD, multiple system atrophy (MSA), or progressive supranuclear palsy (PSP) required agreement among the examiners (neurologists specialized in movement disorders) that the clinical criteria for the diagnosis were fulfilled based on the UK Parkinson's Disease Society Brain Bank criteria^7^ or criteria for MSA or PSP.^8,9^

**Figure 1:**
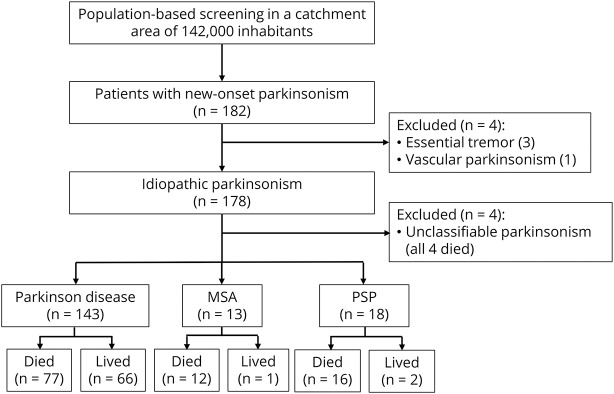
Flowchart of patients included in the study Diagnosis was established according to clinical diagnosis at the latest follow-up and confirmed by autopsy in 5 patients. MSA = multiple system atrophy; PSP = progressive supranuclear palsy.

At the latest follow-up (up to death or 8.5–13.5 years following inclusion in alive patients), 143 patients were diagnosed with PD, 13 with MSA, 18 with PSP, 4 as having unclassifiable parkinsonism, and 4 did not have idiopathic parkinsonism (figure 1). Autopsy confirmed the diagnosis in 3 cases of PD and 2 cases of PSP.

### Standard protocol approvals, registrations, and patient consents

All participants provided written informed consent. The study was approved by the Regional Medical Ethics Board in Umeå, Sweden, and was performed in accord with the Declaration of Helsinki.

### Clinical assessments

All participants were investigated at study entry (baseline) and followed up prospectively with standardized clinical examinations, including the Unified Parkinson's Disease Rating Scale (UPDRS), the modified Hoehn and Yahr Scale, Mini-Mental State Examination (MMSE), and the 39-item Parkinson's Disease Questionnaire, at least yearly, as described previously.^10^ We divided the baseline UPDRS scores into subscores for tremor (sum of items 20 and 21) and postural imbalance and gait disorder (PIGD; sum of items 13, 14, 15, 29, and 30) and classified the phenotype of PD as tremor predominant, intermediate, or PIGD in accordance with the DATATOP (Deprenyl and Tocopherol Antioxidative Therapy of Parkinson’s Disease) trial analyses.^11^ Olfactory function was investigated by the 12-item Brief Smell Identification Test,^12^ global mobility by the Timed Up and Go (TUG) test, which is the time it takes to rise up from a chair, walk 3 m, and sit down again,^13^ and depression by the Montgomery-Åsberg Depression Rating Scale.^14^ Severe hyposmia is defined by a score <4 on the Brief Smell Identification Test. A possible REM sleep behavior disorder was defined by a history of dream enactment behavior on a screening questionnaire.

### CSF analysis

At study entry, 99 of the patients with PD and 21 of the patients with atypical parkinsonism agreed to CSF collection by lumbar puncture, using standard procedures. CSF total tau concentration was measured using a sandwich ELISA (INNOTEST hTAU Ag; Fujirebio, Ghent, Belgium) constructed to determine all tau isoforms irrespective of phosphorylation status.^15^ Tau phosphorylated at threonine 181 was measured by a sandwich ELISA (INNOTEST PHOSPHO-TAU [181P]; Fujirebio).^16^ CSF β-amyloid 1–42 (Aβ_42_) levels were measured using a sandwich ELISA (INNOTEST β-AMYLOID [1–42]; Fujirebio) constructed to measure Aβ containing the first and 42nd amino acids.^17^ CSF levels of α-synuclein were analyzed using a commercially available α-synuclein human ELISA (KHB0061; Invitrogen, Carlsbad, CA) according to instructions by the manufacturer. Experienced, board-certified laboratory technicians performed the CSF analyses using procedures approved by the Swedish Board for Accreditation and Conformity Assessment. Cells in the CSF were counted by automated flow cytometry. A subtle pleocytosis of mononuclear leukocytes was operationally defined by counts ranging between 2 and 20 cells per microliter. However, no patient had more than 10 cells per microliter.

### Dopamine active transporter imaging

Of the 182 patients enrolled in the study, 170 patients (93.4%) underwent dopamine active transporter (DAT) imaging by ^123^I-FP-CIT (DaTSCAN; GE Healthcare BV, Eindhoven, The Netherlands) SPECT. DAT imaging was done 3 hours following an IV bolus dose of 185 MBq ^123^I-FP-CIT. Imaging was done prior to commencement of medication at baseline. The imaging protocol was done within the framework of a nonprofit clinical trial (EU no. 2009-011748-20) and constituted a substudy within the research project. Semiquantitative analysis (based on regions of interest) and visual evaluation of the DAT SPECT were done unbiased by any clinical information at all times. Normal reference values were derived from an age-matched group of healthy controls participating in the study, and reduction of DAT uptake in the patients with PD was measured in percent and SDs of the normal values. The most affected side (left or right) was defined by the putamen and caudate that showed the largest reduction of ^123^I-FP-CIT uptake. The putamen and caudate were investigated separately. The imaging protocol, equipment, and semiquantitative evaluation methods that were used have been described earlier.^18^ Two different SPECT cameras were used during the course of the project; one brain-dedicated SPECT camera (the Neurocam) was later substituted by a multipurpose hybrid SPECT/CT (both General Electric, Milwaukee, WI). Normal reference values were established for both equipments.^18,19^ All PD, MSA, and PSP patients fulfilling diagnostic criteria and who participated in the DAT imaging (n = 163) had a pathologic scan.

### Genetic testing

One hundred thirty-three of the patients with PD agreed to DNA analysis by peripheral blood sampling. DNA was isolated from peripheral blood using standard procedures. The variations of interest (the ε2/ε3/ε4 polymorphisms) in the *APOE* gene were genotyped using TaqMan Assays-by-Design (Applied Biosystems, Foster City, CA). The assay was performed according to manufacturer's instructions and analyzed using the allelic discrimination function of the TaqMan 7900 HT Fast Real-Time PCR system (Applied Biosystems). Genotype success rates of 100% were obtained. The patients who declined all laboratory investigations (n = 10) were older than the others (79.1 vs 70.5 years) and had slightly higher UPDRS scores for motor dysfunction but were otherwise comparable. All laboratory analyses were performed blinded from clinical data.

### Neuropsychology

Extensive neuropsychological testing, used for PD-MCI and PDD^20^ diagnostics, was performed at baseline and after 1, 3, 5, and 8 years. At baseline, the patients with PD could be divided into patients with MCI (PD-MCI, n = 61) and patients without PD-MCI (PD with normal cognition, n = 82). Because all cognitive domains had been covered in the test battery throughout the study period, PD-MCI diagnoses were applied according to Movement Disorder Society guidelines,^5^ using level 2 criteria.^21^ Patients were classified as having MCI at baseline if (1) impaired in a minimum of 2 tests in one domain (single-domain MCI) or in a minimum of one test in 2 different domains (multiple-domain MCI), (2) impairments were ≥1.5 SDs below mean of normative data, (3) self-perceived cognitive decline was reported in a questionnaire (e.g., the 39-item Parkinson's Disease Questionnaire) and/or directly by patient and/or family member, and (4) the patient had no functional impairment in basic activities of living (i.e., driving a car, social or personal care, medication management) due to cognitive impairment. The tests used for MCI classification were, for episodic memory: Free and Cued Selective Reminding Test, Logical Memory and Paired Associative Learning from the Wechsler Memory Scale, and Brief Visuospatial Memory Test (total recall); for working memory: Digit Span Forward and Digit Span Backward from Wechsler Adult Intelligence Scale III; for attention: Trail Making Test A and B; for language: Controlled Oral Word Association and Boston Naming Test; for visuospatial function: the Benton Judgment of Line Orientation Test and Pentagon Copying from MMSE; and for executive function: Wisconsin Card Sorting Test–computer version 2, Mental Control from Wechsler Memory Scale, and Category Fluency (animals in 60 seconds). In the multivariable analysis of factors predicting mortality in PD, baseline neuropsychology results were used. However, to investigate possible effects of incident dementia (of which there were no cases at baseline), the neuropsychology results from the 3-year follow-up were also analyzed in relation to mortality. Incident PDD and PD-MCI were diagnosed at the 3-year follow-up on the basis of previous test results, a documented decline, cognitive decline reported by the patient and/or family member, and (for PDD) by the occurrence of functional impairment in basic activities of living caused by cognitive impairment. Structural MRI and routine laboratory tests were performed to exclude cognitive impairment due to other causes than PD-MCI or PDD.

### Mortality

We followed all surviving patients yearly for approximately 8.5 to 13.5 years, until August 31, 2017. The average time since inclusion in all patients was 7.7 years. Although a few older patients were followed by telephone, rather than visits, during the last few years, the survival data were complete. A death certificate, in which the cause of death was stated, was obtained for 98 (90%) of all the fatalities in the cohort.

### Statistics

Potential group differences in clinical variables at baseline were examined by 1-way analysis of variance, Fisher exact test, and Kruskal-Wallis test, and correlation between variables by Person *r* and Spearman ρ, as appropriate. Post hoc contrasts in analysis of variance are shown corrected for multiple comparisons using Tukey HSD (honestly significant difference). The age- and sex-specific standardized mortality ratio (SMR) was calculated, stratified by clinical diagnosis and sex and, in the patients with PD, by MCI status, by dividing the observed number of deaths counted from baseline in each group by the expected number of deaths in each group. The expected numbers of deaths were calculated using the age- and sex-specific official Swedish National Statistics of 2004–2017 mortalities multiplied by the person-time from each group in the study. Confidence intervals (CIs) for the SMRs were calculated to the 95% level using the Poisson distribution and *p* values using the χ^2^ distribution. Life expectancies (anticipated mean remaining time to live) based on SMR were calculated using a modified Gompertz function, as done previously.^22^ Cox proportional hazard analysis was used to investigate whether one or more covariates predicted mortality in PD. A list of potential risk factors for mortality was generated, including factors previously found predictive and factors of neurobiological interest (biomarkers). To investigate dopaminergic denervation at baseline, DAT-uptake ratios were normalized by average SDs above or below the normal mean (i.e., *z* score) in order to equate the numerical values derived from the 2 different scanners that were used. The number of mononuclear leukocytes in CSF was investigated as a continuous predictor variable in relation to survival. The presence of a CSF leukocytosis was also investigated. For univariate comparisons, we controlled for a false discovery rate of 0.05 by using the Benjamini-Hochberg procedure, resulting in a significance level of *p* < 0.017. Because age is strongly related to survival, all results were adjusted for age. Multivariable models were then developed using age with clinical variables and age with biomarker variables as predictors of survival. Variables significantly associated with survival at the *p* < 0.2 level in the univariate models were included, using a backward elimination procedure. If variables were highly correlated, the variable with the lower *p* value was included to avoid multicollinearity. The only exception was the PIGD score, which was correlated to TUG results but chosen over the TUG test because of perceived higher clinical usefulness. In the final multivariable models, only variables with *p* < 0.005 were included to avoid overfitting of the model and to restrict the number of predictors for ease of use in clinical practice. To avoid confounding related to the heterogeneity of the different parkinsonian diseases, detailed prognostic models were developed only for PD, except for the CSF protein markers, which were also investigated in atypical parkinsonism. All statistical analyses were performed using SPSS 23.0 (IBM Corp., Armonk, NY) or R Statistical Software.

### Data availability

Anonymized data can be obtained by request from any qualified investigator for purposes of replicating procedures and results.

## Results

### Survival in incident idiopathic parkinsonism

Clinical characteristics at baseline for the patients with idiopathic parkinsonism are shown in table 1. Survival data from first evaluation to death or end of the study were obtained for all participants (figure 1). Of the 178 patients with idiopathic parkinsonism, 109 (61.2%) died during follow-up. Seventy-seven (53.8% of 143) of the deaths occurred in the PD group, 12 (92.3% of 13) in the MSA group, and 16 (88.9% of 18) in the PSP group. The 4 patients with unclassifiable parkinsonism likely represent cases of late-onset PD but were excluded from further analyses, as they did not fulfill specific diagnostic criteria. The overall mean age at death was 82.0 years. Deep brain stimulation or pumps for intestinal delivery of levodopa were used or had been used by 12 (8.4%) of the 143 patients with PD.

**Table 1:**
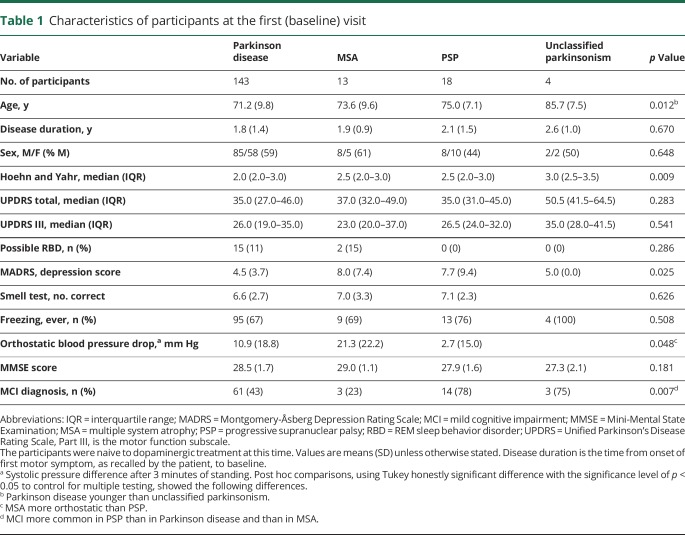
Characteristics of participants at the first (baseline) visit

Survival was related to the age at the first visit (hazard ratio for death: 3.03 for each decade older, 95% CI 2.30–3.98, *p* < 0.001). However, when survival in patients with MSA or PSP was analyzed separately from PD, the age at the first visit was not a significant factor (*p* = 0.766). As a measurement of global cognitive function, survival also correlated with the baseline MMSE score after adjustment for age (1.19 times higher hazard for death for each lower point, *p* = 0.006). The SMR for the whole parkinsonism cohort was 1.84 (*p* < 0.001) times higher than the comparable age and sex distribution-standardized mortality of the Swedish population during the years 2004 to 2017 (table 2).

**Table 2:**
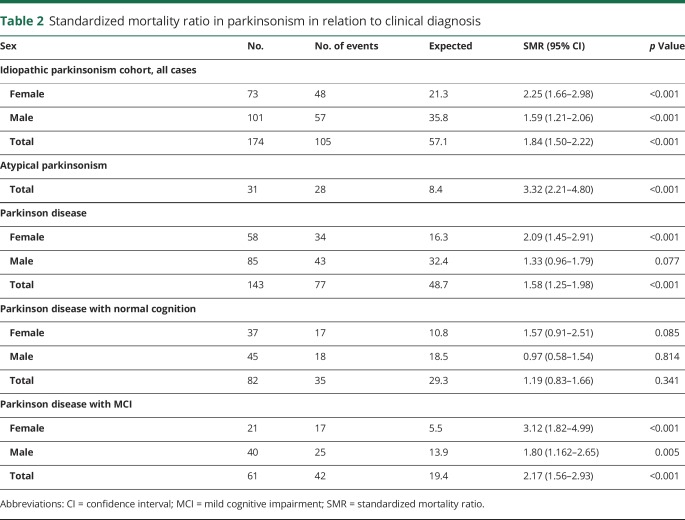
Standardized mortality ratio in parkinsonism in relation to clinical diagnosis

**Figure 2:**
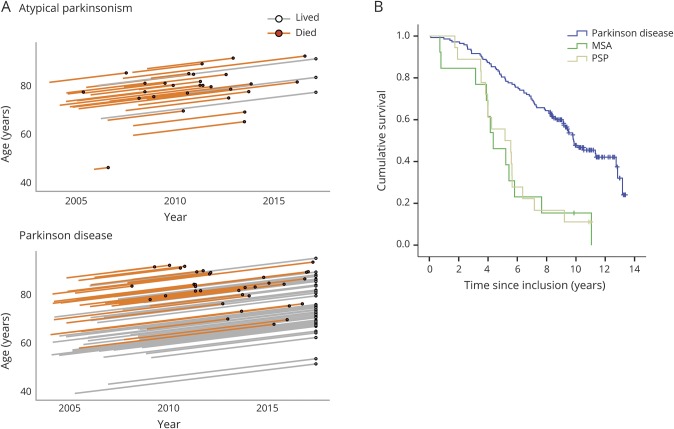
Survival in incident, idiopathic parkinsonism (A) Lexis diagram showing follow-up of patients throughout the study. The “atypical parkinsonism” group comprises patients with new-onset MSA and PSP. (B) Kaplan-Meier plot of survival in relation to diagnosis (for number at risk, see supplemental table e-1, links.lww.com/WNL/A762). MSA = multiple system atrophy; PSP = progressive supranuclear palsy.

The SMR for the patients with PD was 1.58 and 3.32 for the patients with atypical parkinsonism (table 2). The patients with PD had an age-adjusted hazard ratio for death of 0.43 (95% CI 0.27–0.67, *p* < 0.001) compared to the patients with atypical parkinsonism. More specifically, the age-adjusted hazard ratio for death in MSA was 2.76 (95% CI 1.48–5.15, *p* = 0.001) and 1.42 (95% CI 1.08–1.88, *p* = 0.012) in PSP compared to the patients with PD (figure 2). As shown in table 3, the most common cause of death was pneumonia, but in many cases, the exact cause of death could not be determined (e.g., patients who died alone in their homes). Assuming the mean age of 71.7 years at baseline in idiopathic parkinsonism, the expected survival in PD was 9.6 years, and 6.1 years in atypical parkinsonism.

**Table 3:**
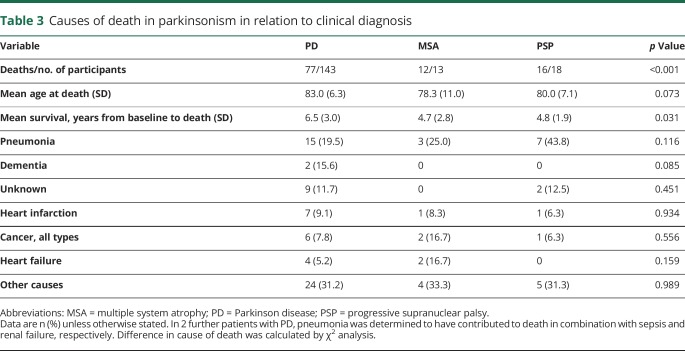
Causes of death in parkinsonism in relation to clinical diagnosis

### Survival in PD

Although there was no sex difference in the hazard ratio for death in the patients with PD, the SMR was higher in female (2.09, i.e., 109% more cases of death in female patients with PD than in the general female Swedish population) than in male (SMR: 1.33) patients (table 2). The survival also correlated with cognitive status. Mortality in patients with PD (both male and female) with normal cognition at baseline was not significantly different from the general Swedish population, while it was increased in patients with PD-MCI (SMR: 2.17; table 2). The patients with PD-MCI at baseline had a 2.4 times higher age-adjusted hazard of death during follow-up compared to the patients without MCI at baseline (*p* < 0.001, table 4). Survival in PD also correlated with the baseline MMSE score (1.17 times higher hazard for death for each lower point, after adjustment for age, *p* = 0.011). Fewer years of formal education was not associated with reduced survival. Assuming the mean age of 71.2 years at baseline, the expected survival in PD was 11.6 years without MCI and 8.2 years with MCI.

**Table 4:**
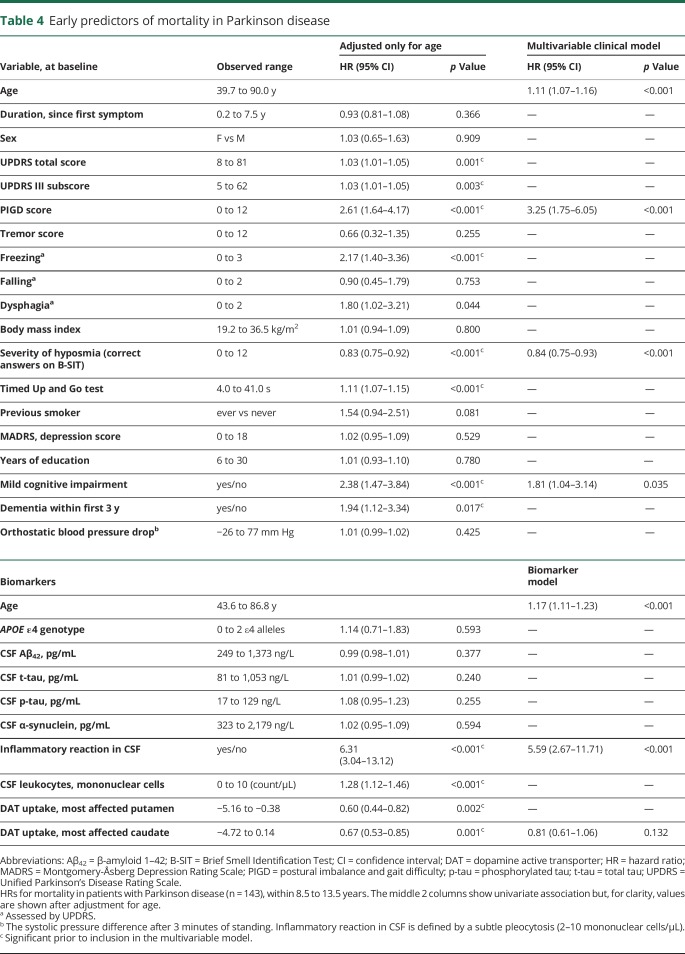
Early predictors of mortality in Parkinson disease

**Figure 3:**
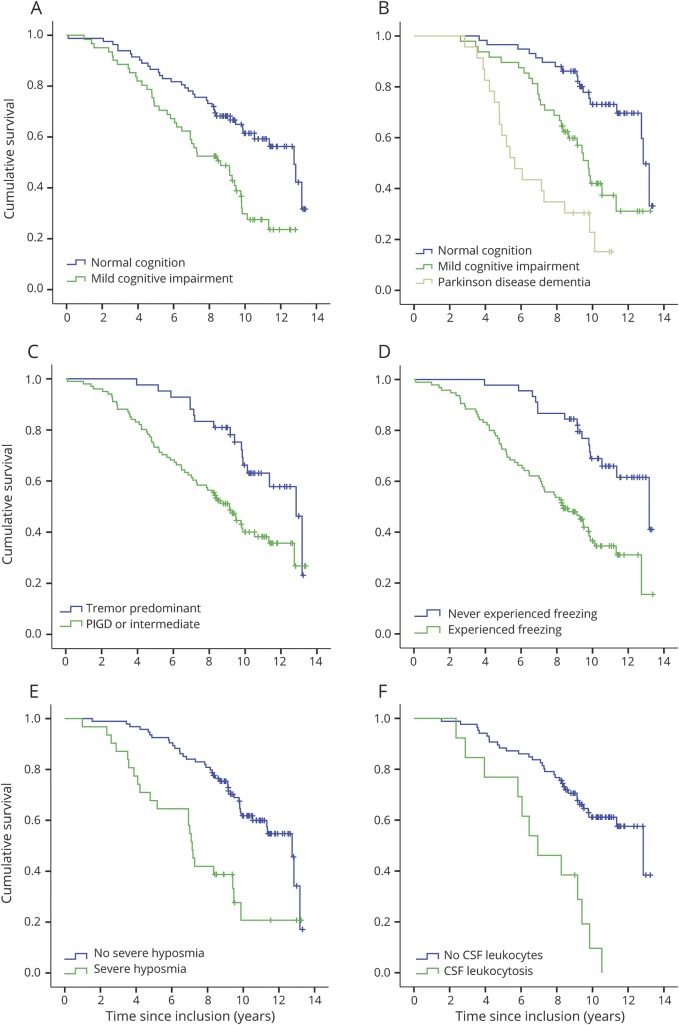
Survival in Parkinson disease in relation to phenotype Kaplan-Meier plots of survival in patients with Parkinson disease (n = 143) in relation to clinical and neurobiological phenotype at baseline (except panel B, which is related to phenotype at 3 years). Severe hyposmia is defined by a B-SIT score <4. All variables (A–F) were significantly related to survival at the *p* < 0.001 level (log rank) except the tremor or PIGD/intermediate variable (C), which was significant at the *p* = 0.004 level (for number at risk, see supplemental table e-2, links.lww.com/WNL/A762). B-SIT = Brief Smell Identification Test; PIGD = postural imbalance and gait disorder.

A baseline PD phenotype comprising MCI, freezing of gait, a PIGD phenotype, hyposmia, high disease severity (measured by the total and Part III UPDRS scores), slowness in the TUG test, and onset of dementia during the first 3 years predicted shorter survival (table 4 and figure 3, A–F) in the univariate analysis. Tremor was uncorrelated to survival. The patients with PD had a 38% lower DAT uptake in the putamen and a 24% lower DAT uptake in the caudate nucleus in the most affected hemisphere compared to healthy controls. A neurobiological factor (biomarker) that predicted a shorter survival in PD was reduced DAT uptake in the striatum, particularly in the caudate nucleus (1.48 times higher hazard for death for each SD of lower uptake), while the baseline CSF protein concentrations of neurodegenerative markers and *APOE* genotype were not predictive (table 4). Higher Aβ_42_ concentration in CSF was, however, moderately associated with increased survival when the whole cohort of patients with parkinsonian disorders was investigated (age-adjusted hazard ratio: 0.99 per pg/mL, 95% CI 0.98–1.00, *p* = 0.048 in all patients and 0.97 per pg/mL, 95% CI 0.95–1.00, *p* = 0.036 in the patients with atypical parkinsonism). Furthermore, a proportion of all patients with PD (13.1%) had a subtle pleocytosis of mononuclear leukocytes (2–10 cells) in the CSF. These patients had a significantly worse prognosis compared to the other patients (hazard ratio for death: 6.31, *p* < 0.001; table 4), independently of adjustment for erythrocytes. Given this finding, the CSF results were further analyzed. There was a trend that a higher number of leukocytes at baseline tended to correlate with higher CSF total tau and phosphorylated tau concentrations (*r* = 0.24, *p* = 0.019 and *r* = 0.22, *p* = 0.028, respectively). There was no evidence of CNS infection in any of the patients and there was no difference in neurodegenerative marker or total protein concentrations in the CSF between PD patients with a leukocytosis and the other PD patients. However, the PD patients with a CSF leukocytosis had a higher progression of the UPDRS III scores at the 1-year follow-up (median 4-point increase vs 3-point decrease, *p* = 0.007, Mann-Whitney *U* test) than the other patients with PD, despite using equal medication dosages (as measured by levodopa-equivalent daily dose).

After excluding factors strongly correlated to each other and including only strictly significant factors (at the *p* < 0.005 level) in a multivariable model, older age at baseline, prevalent mild cognitive impairment, higher PIGD score, hyposmia, reduced DAT uptake in the caudate, and an inflammatory reaction (leukocytosis) in the CSF were independent clinical and biomarker predictors of reduced survival in PD.

## Discussion

In this prospective, population-based cohort study involving in-person examination of all participants, we evaluated the prognosis of idiopathic parkinsonism and PD with respect to mortality, stratified by factors related to MCI and clinical and neurobiological characteristics. To avoid selection bias, we included all patients with incident idiopathic parkinsonism in the studied area, rather than only PD, and we explicitly diagnosed atypical forms of parkinsonism. Our results should, therefore, provide information that is generalizable to the “real-life” experience of the overall population with idiopathic parkinsonism.

In line with the findings from several previous studies,^23,24^ patients with MSA and PSP had a markedly worse prognosis than patients with PD. In the present cohort, the patients with incident, atypical parkinsonism (i.e., MSA or PSP) were older than in most previous, hospital-based studies,^24–26^ possibly because of the population-based design. Our results confirm the dire prognosis of these diseases in this cohort, with a relatively high observed age at onset. In fact, age was not a significant predictor of survival in patients with MSA or PSP, which is likely explained by the strong effect on mortality caused by these disorders themselves.

Recent studies have also, despite advances in modern treatment, found a reduced life expectancy in PD.^1^ Similarly, we found that the survival of patients with PD was reduced in comparison with the general Swedish population during the same years (SMR 1.58, *p* < 0.001). The SMR in most modern PD mortality studies has been in the range of 1.5 to 2.7.^1,2^ Hence, the risk of death in the present study was in the lower range. This may be a result of the population-based study protocol (including patients with mild parkinsonism and explicitly diagnosing cases of atypical parkinsonism, that have a worse prognosis) and the fact that all patients had access to comprehensive health care services throughout the disease course.

It is currently unclear why many patients with PD experience shorter lifespans. In the present PD cohort, the excess mortality occurred most clearly in female patients and in patients with MCI (with pneumonia being the most common cause of death). Data on the general Swedish population show longer survival in women than in men. Therefore, a similar death rate (i.e., a hazard ratio close to 1) between the sexes in the present study, as well as in previous studies,^27,28^ may be interpreted to indicate a worse than expected prognosis for female patients with PD. In keeping with this, some previous PD studies have explicitly reported a higher mortality among female patients.^29–31^

The fact that life expectancy is reduced in PD compared to the general population independently from comorbidities,^32^ and the finding of a correlation between mortality and severity of PD symptoms as measured by clinical scales,^33,34^ suggests that disease-specific features (such as α-synuclein pathology) at least partly account for the increased mortality. We found that the increased mortality in PD correlated with core parkinsonian symptoms (with the notable exception of tremor) and olfactory dysfunction, independently of age. The elevated mortality in PD also correlated with the severity of striatal DAT imaging deficits, both in the putamen and in the caudate. Our findings indicate that the neurodegenerative process in PD, which is linked to nigrostriatal denervation, is in itself associated with increased mortality. Of note, resting tremor in PD has been found to only weakly correlate with striatal dopaminergic denervation.^35^

In contrast, mortality was not increased in patients with PD who did not have cognitive impairment at study entry (male patients without MCI actually had an SMR below 1). The findings underscore the importance of an early PD-MCI diagnosis, as defined by the Movement Disorders Society. There is evidence that PD is associated with lower risk of some diseases, such as many cancers, and a lower risk factor burden from tobacco smoking and arterial hypertension.^36–39^ In PD patients with normal cognition, who have a milder disease phenotype, such differences could counterbalance mortality increases caused by neurodegeneration. Furthermore, a lower level of education was not associated with increased mortality. This may indicate that the higher mortality in patients with poorer cognitive function was related to the pathologic process leading to cognitive dysfunction, rather than being explained by socioeconomic factors. In keeping with this interpretation, a few previous studies have found dementia in PD to be an independent risk factor for death.^3,40^ Of note, we found higher mortality in PD patients with more severe caudal DAT-uptake deficits. Imaging studies (including an fMRI study of the same patients with PD as those in the present study) have shown that dopamine denervation in the caudate nucleus in PD correlates with cognitive deficits.^41,42^

A positive *APOE* ε4 carrier status or α-synuclein, Aβ_42_, and total tau or phosphorylated tau concentrations in CSF were uncorrelated with PD mortality. However, the number of CSF samples may have made our study underpowered for detecting such differences. The difference compared to the findings in a recent neuropathologic study^43^ could also relate to the fact that the present investigation used a more homogeneous disease group (PD). When CSF samples from all patients with idiopathic parkinsonism were included, a lower Aβ_42_ concentration was associated with shorter survival. This may indicate that Alzheimer disease–type pathology is a predictor of reduced survival in parkinsonian disorders in general,^43^ mainly in MSA and/or PSP.

The finding of a low-grade inflammatory reaction in the CSF of 13.1% of the patients with PD was strongly related to a reduced survival (with a 6.31 times increased hazard for death). An increase of proinflammatory cytokines in the CNS and an inflammatory, hyperreactive state in circulating monocytes has previously been shown in PD.^44,45^ Two large, observational studies have also found a lower risk of PD associated with the use of nonsteroidal anti-inflammatory drugs in the general population.^46,47^ Taken together, our findings might suggest a triggered immune system, responding to the presence of abnormal, misfolded proteins in PD patients with short lifespans, possibly contributing to disease progression. The shorter lifespans in patients with a CSF leukocytosis indicate a rationale for further investigating immunomodulation to reduce PD mortality.

The possibility of uncontrolled confounding factors and the fact that neuropathologic diagnosis at autopsy of the nervous system was obtained in only 5 of 109 deaths are limitations of the study. Several studies have reported that neuropathologic confirmation of a clinical diagnosis of idiopathic PD ranges from 65% to 93%,^48^ although the accuracy is higher in expert centers.^49^ In addition, no autopsy examinations were performed in any of the MSA cases. While acknowledging the limitations of a clinical diagnosis, the risk of incorrect diagnosis was nonetheless minimized by the long follow-up periods and the finding of pathologic uptake on DAT imaging in all of the examined patients. Even so, the results, especially the CSF findings, need to be confirmed by future studies. Our study also has several strengths, including a population-based design, a high proportion of patients who were examined using a multimodal research protocol, and prospective follow-up for up to 13.5 years.

The present study shows that patients with incident parkinsonism have reduced survival but that the survival is highly dependent on the type and characteristics of the parkinsonian disorder. Early MCI in PD is an important predictor of the prognosis. The finding of a low-grade immune reaction in the CSF of patients with PD who have short survival may have important clinical implications and therefore merits further investigation.

## Publication history

Received by *Neurology* April 16, 2018. Accepted in final form August 15, 2018.

## Glossary

Aβ_42_: β-amyloid 1–42
CI: confidence interval
DAT: dopamine active transporter
MCI: mild cognitive impairment
MMSE: Mini-Mental State Examination
MSA: multiple system atrophy
PD: Parkinson disease
PDD: Parkinson disease dementia
PIGD: postural imbalance and gait disorder
PSP: progressive supranuclear palsy
SMR: standardized mortality ratio
TUG: Timed Up and Go
UPDRS: Unified Parkinson's Disease Rating Scale
